# HANDY: a device for assessing resistance to mechanical crushing of maize kernel

**DOI:** 10.1186/s13007-021-00729-2

**Published:** 2021-04-26

**Authors:** Yuan Su, Yang Xu, Tao Cui, Xiaojun Gao, Guoyi Xia, Yibo Li, Mengmeng Qiao, Yingbo Yu

**Affiliations:** College of Engineering, China Agricultural University, NO. 17 Qinghua East Road, Beijing, 100083 People’s Republic of China

**Keywords:** Maize kernel, Crushing resistance, Breakage susceptibility test, HANDY

## Abstract

**Background:**

How to control the physical damage during maize kernel harvesting is a major problem for both mechanical designers and plant breeders. A limitation of addressing this problem is lacking a reliable method for assessing the relation between kernel damage susceptibility and threshing quality. The design, construction, and testing of a portable tool called “HANDY”, which can assess the resistance to mechanical crushing in maize kernel. HANDY can impact the kernel with a special accelerator at a given rotating speed and then cause measurable damage to the kernel. These factors are varied to determine the ideal parameters for operating the HANDY.

**Results:**

Breakage index (BI, target index of HANDY), decreased as the moisture content of kernel increased or the rotating speed decreased within the tested range. Furthermore, the HANDY exhibited a greater sensitivity in testing kernels at higher moisture level influence on the susceptibility of damage kernel than that in Breakage Susceptibility tests, particularly when the centrifugation speed is about 1800 r/min and the centrifugal disc type is curved. Considering that the mechanical properties of kernels vary greatly as the moisture content changes, a subsection linear (average goodness of fit is 0.9) to predict the threshing quality is built by piecewise function analysis, which is divided by kernel moisture. Specifically, threshing quality is regarded as a function of the measured result of the HANDY. Five maize cultivars are identified with higher damage resistance among 21 tested candidate varieties.

**Conclusions:**

The HANDY provides a quantitative assessment of the mechanical crushing resistance of maize kernel. The BI is demonstrated to be a more robust index than breakage susceptibility (BS) when evaluating threshing quality in harvesting in terms of both reliability and accuracy. This study also offers a new perspective for evaluating the mechanical crushing resistance of grains and provides technical support for breeding and screening maize varieties that are suitable for mechanical harvesting.

**Supplementary Information:**

The online version contains supplementary material available at 10.1186/s13007-021-00729-2.

## Background

The maize has the highest yield compared with other food crops. The planting area of maize was more than 41,284 hectares and the total yield was more than 1108.6 million tons in 2019 in China (National Bureau of Statistics [1]).

Remarkably, the serious physical damage of maize kernels caused by mechanical processing has become the primary factor that can affect the quality and grand of maize kernels [2]. On the one side, if maize seeds have stronger crushing resistance, it can get a higher completeness probability in mechanical sowing. This can also effectively improve the germination rate of seeds and grain yield. It is estimated that Chinese farmers lose almost 247.5 kg/ha per year in maize yield due to mechanical damage of kernels in harvesting [3]. On the other side, the safety of grain storage has always been the focus of relevant researchers. The undamaged kernels are also less prone to mildew and pests, leading to deterioration during storage. Tracing to the source, to meet the needs of practical applications, screening and developing damage-resistant cultivars are the most cost-effective and secures means, it brings a great challenge to the breeders.

Therefore, increasing the mechanical crushing resistance of maize kernels is important to both current food security and the development of maize varieties [4]. Hence, a study on increasing impact strength of maize kernel has important significance to develop commercial harvesters and enhance maize quality grade.

Maize kernel crushing resistance is a key determinant of the threshing quality as it can affect the required capability for keeping the integrity of kernels. Various testing methodologies for predicting the crushing resistance of maize kernels have been presented, the methodologies include the compression method [5, 6], drop method [7], pendulum method [8] and breakage susceptibility method [9, 10]. Besides, several researchers have sought to establish correlations between various morphological [11], chemical [12, 13], mechanical properties [14, 15], genetic [16], or environmental [17] factors of maize kernels and breakage susceptibility (e.g., measurements of density, hardness, protein content, etc.). Unfortunately, the coefficient of determinations of the regression equations is unsatisfactory, or the results lack in making a comparative discussion from the threshing aspect [14].

Furthermore, these methods are typically labor-intensive and often require expensive laboratory equipment. For instance, laboratory-based compression and puncture tests are completed by a universal testing machine, which needs to be repeated a dozen times and take the average value [18–21]. Besides, some of these methods do not produce the same damage types observed in mechanical threshing maize. Specifically, the predominant damage type of maize kernels in these test methods is distinctive cracks but that in mechanical threshing is fragments [14].

This study describes a portable tool called “HANDY” for assessing the mechanical crushing resistance of maize kernels. The design principle of HANDY refers to the form of being loaded of maize kernel in actual threshing. The load in threshing is mainly impacted forces, thus, the device uses centrifugal acceleration for imparting impact forces to kernels. The centrifugal acceleration is achieved by a centrifugal disc. Furthermore, the different peripheral speeds and types of the centrifugal disc are used to generate different levels and directions of impact forces, the combination of peripheral speeds and centrifugal disc types are studied to determine the ideal working parameters. Finally, a model to predict the threshing quality derived from the measured result of the HANDY. From the farmers’ perspective, it also provides an effective reference for appointing an opportune harvest time to decrease harvesting losses. Biologically, lacking such a device has been a crucial limitation for breeding efforts focused on suitability for mechanical harvest.

## Description of HANDY

### Structure of HANDY

**Fig.1:**
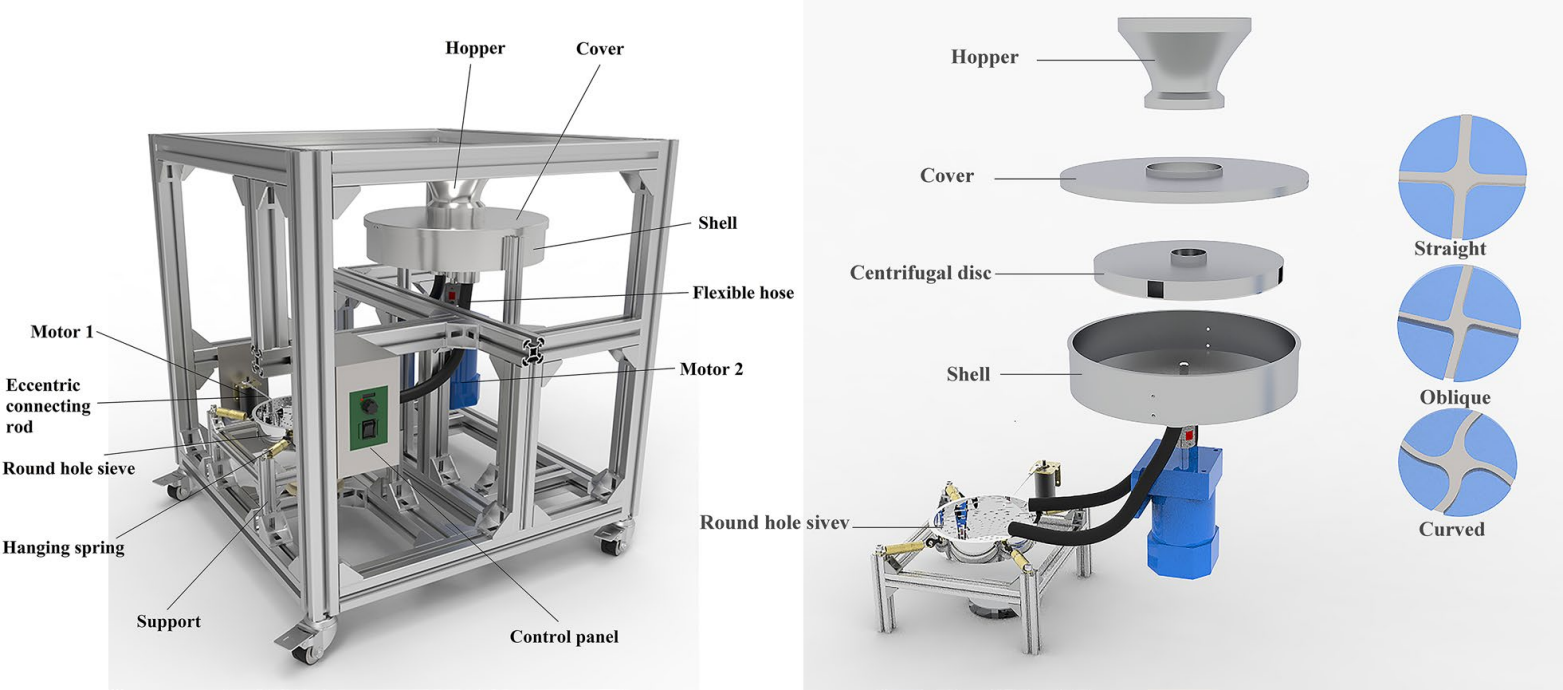
Modified crush resistance tester

The crushing resistance impactor apparatus (HANDY), as shown in Fig. 1, is composed of an impact part, a sieve part and a frame part (Additional file 1). The impact part includes a hopper, a cover, a shell, a centrifugal disc and a motor, and they are arranged concentrically from top to bottom. A material feeding plate is set under the hopper. The hopper is mounted on the feed port which is set on the top surface of the cover. The cover and the shell are tightly fixed by four buckles. A centrifugal disc is installed inside the shell, and directly driven by motor 2. Note that the centrifugal disc is composed of a disc plate and a transmission shaft. Six triangular side plates are welded to the disc plate and the shaft to maintain the stability of the rotation of the centrifugal disc. The shaft of motor 2 transfers the torque through the coupling to the centrifugal plate. To make the kernels flow down smoothly, the lower part of the shell is conical, and two symmetrical discharge ports are designed and set at the bottom of the shell (Additional file 2).

The sieve part is composed of a sieving mechanism and a driving mechanism. The sieving mechanism is composed of a sieve frame and a round hole sieve. The round hole sieve is connected with the frame that is composed of four columns, and the connection is achieved by four suspension springs. The driving mechanism is composed of motor 1 and the crank connecting rod mechanism. Motor 1 is the power source that can drive the crankshaft and connecting rod mechanism, then, it forms the reciprocating motion of the sieve. Two bendable pipes are used to convey samples from the impact part to the sieve part. The upper end of the pipes is fixed with the discharging ports. The lower end of the pipes is set above the sieve.

### Working mechanisms of HANDY

First, placing a sample of maize kernels in the hopper. Then energizing the motors. Driven by motor 2, the centrifugal disc will rotate at a certain speed. The speed of motor 2 is set by using a variable speed drive. Then drawing out the feeding plate. Under the action of gravity force, the kernels will fall into the feeding port. Under the action of centrifugal force, the kernels will be accelerated rapidly by the centrifugal disc, and be thrown out of the centrifugal disc at a certain speed. Then, kernels will collide with the inner wall of the shell. After that, all the kernels will fall into the bottom of the shell and then slide into the sieve through the pipes. Finally, the fragment kernels will be sieved by the sieve under a certain vibration frequency.

## Testing of HANDY

### Purpose of the tests

The first purpose of the tests is to determine the optimum parameters for the HANDY. The centrifugal speed and type of centrifugal disc are chosen because they can affect the levels and directions of impact forces that are applied to kernels. The levels and directions of impact forces can further influence the repeatability and uniformity of the results. Moreover, if the system is operated at an unsuitable speed, it will erratically produce vibration which is unstable for experimental purposes.

The second purpose of the tests is to access the performance of HANDY, which is discussed by comparing the test result of the HANDY to the Breakage Susceptibility test. Note that the Breakage Susceptibility test is commercially used to evaluate the mechanical strength of the kernel, which is operated by an acceleration device [22].

The third purpose of the test is to indicate that the HANDY can be used to predict the threshing quality. The method is to build a model to show the relationship between the BI (the crushing resistance of maize kernels assessed by the HANDY) and its broken rate (BR, the index of threshing quality) in actual mechanical threshing.

### Procedure of the testing

For the Breakage Susceptibility test, the HANDY test and the mechanical threshing test, 21 commercial common maize hybrids from northern China are utilized as the test material (Additional file 3). The moisture content of the kernels is determined (15.8–30.9%) using a grain moisture measurement instrument (Japan, KETT, PM–8188–A).

For the Breakage Susceptibility test and the HANDY test, the sample maize kernels are threshed manually and are cleaned to remove all foreign materials, such as dust, female flower and damaged kernels. After that, the kernels of the same variety are mixed evenly, 200 g sample is set as a sample group, and weighed by an electromechanical counter (with an accuracy of 0.01 g) and then poured into the hopper for the tests. The replicants of tests are set as five.

**Fig.2:**
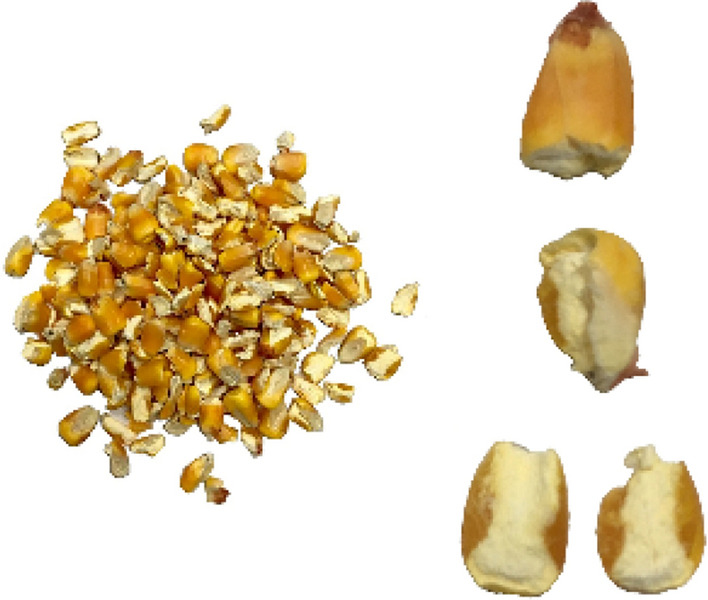
Type of breakage kernels caused by crush resistance tester

For the HANDY test, the breakage index (BI), is the ratio of the weight of all completely crushed kernels (without seed coat connection) to the total sample (as shown in Eq. 1). Note that the crushed kernels are composed of two parts: sieved and un-sieved broken kernels. The un-sieved broken kernels are picked manually and their characteristics are shown in Fig. 2.

1 $$BI = \frac{{W_{S} + W_{US} }}{{W_{1} }} \times 100\%$$

where *W*_*s*_ refers to the weight of the sieved broken kernels, g; *W*_*us*_ refers to the weight of broken kernels that un-sieved g; *W*_*1*_ is the total weight of a set of samples, g.

For the Breakage Susceptibility test, the Breakage susceptibility (BS) of the samples is determined using the HANDY with the traditional straight centrifugal discs. A feed rate of 100 g/min and a centrifugal speed of 27 m/s are used in the test. BS is characterized by the ratio of the weight of the sieved broken kernels to the total samples (as shown in Eq. 2). Note that the tested samples are sieved through12/64-inch openings.

2 $$BS = \frac{{W_{S} }}{{W_{1} }} \times 100\%$$

**Fig.3:**
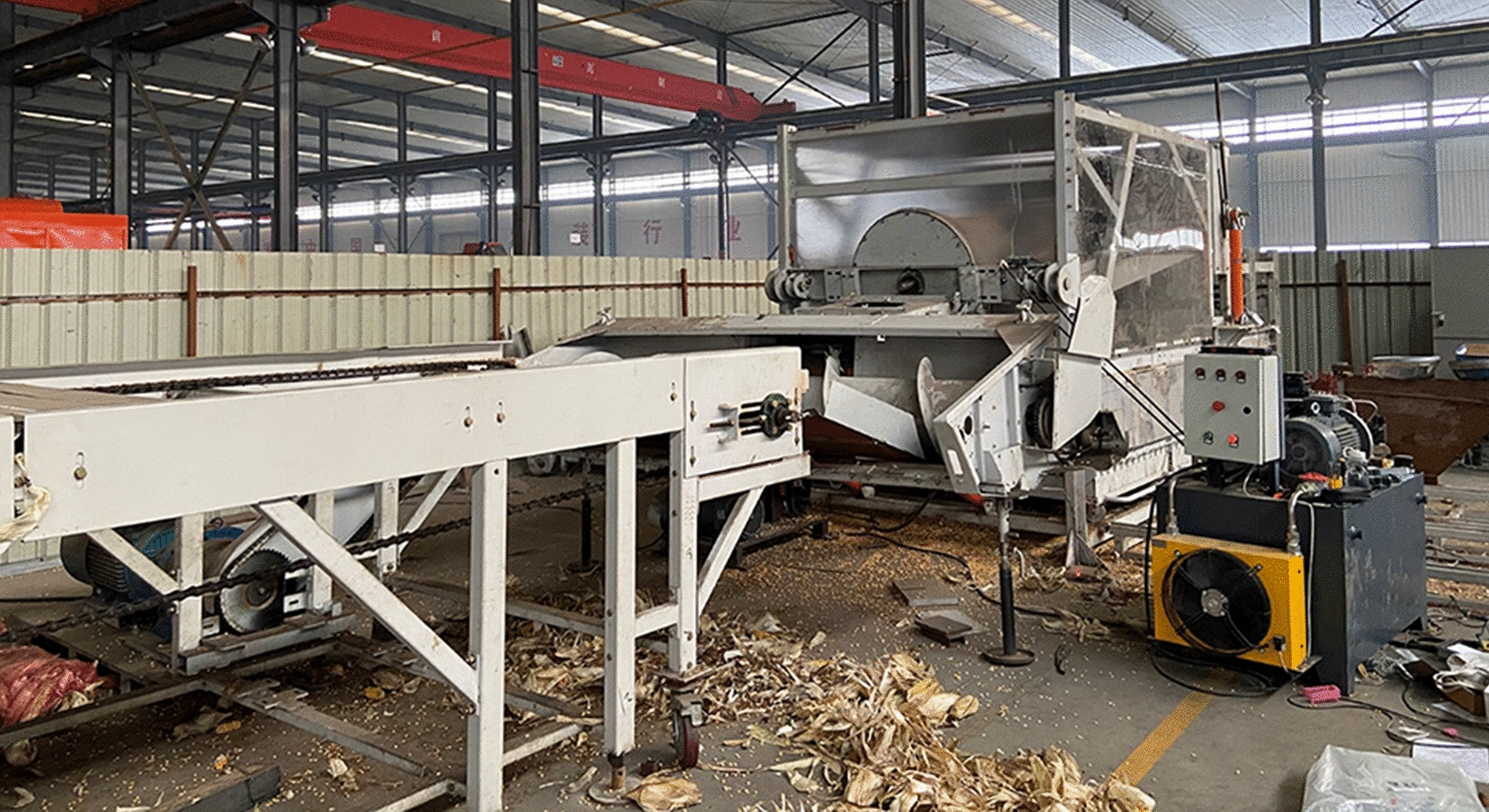
Threshing device of device

For the mechanical threshing test, the whole maize ear is utilized. An axial flow corn threshing cylinder (rotating speed is 300 r/min, concave clearance is 55 mm) is used to thresh maize ears (Fig. 3). The type of threshing element is the cylinder chose rasp bars. The threshing cylinder is designed with a diameter of 520 mm and a length of 2700 mm.

In each experiment, the feeding rate of the threshing cylinder is 8 kg/s. The experiment for each group is repeated three times. Note that the broken rate (BR) of the kernel is an important index to evaluate the threshing quality and separator device [23]. The BR is calculated as follows:

3 $$BR = \frac{{W_{m} }}{{W_{2} }} \times 100\%$$

where *W*_*m*_ refers to the mass of the weight of the broken kernels that have an obvious broken characteristic, g; *W*_*2*_ refers to the total mass of a set of samples for mechanical threshing test, g.

### Baseline testing of HANDY

Baseline experiments are conducted to determine the experimental variables that could be tested and then determine the optimum parameters for operating the HANDY. The baseline experiments are accompanied by using the two varieties (SR 999, ZD 958) of maize samples. For SR 999 and ZD 958, the moisture content of the kernel of 25.32 and 24.75%, respectively. The type of centrifugal disc is straight. The rotating speeds of the disc are 1300, 1500, 1800, 2100 and 2300 r/min (corresponding to 20.4, 23.6, and 26.7, 28.3, 33 and 36.1 m/s peripheral speed).

The basic procedure of the baseline test is to impact a set of samples at a certain rotating speed for a certain amount of time, stop the impact, collect the resulting mixture. The test times mean the resulting mixture is loaded back into the hopper and the process is repeated once, twice, and third. The test times are not the number of repetitions of the test. The number of repetitions of the test means that the same experiment needs to be repeated several times, and taking the average value. The BI obtained for each centrifugal speed and the number of impacts for the two maize varieties, are shown in Table 1.

**Table 1:** BI (%) obtained in the baseline study at the kernel moisture content of 25%

	SR 999			ZD 958		
Peripheral speed/rpm	Once	Twice	Three	Once	Twice	Three
1300	0.19	0.23	0.25	0.21	0.25	0.26
1500	0.61	0.93	1.01	0.52	0.99	1.04
1800	4.25	6.97	7.54	5.71	8.75	9.91
2100	19.69	27.42	28.93	21.41	29.34	31.03
2300	29.25	39.83	41.50	28.35	40.86	42.26

As shown in Table 1, for both test maize varieties, the increase of the BI is negligible when the kernels are repeatedly impacted twice and three times. Besides, repeated impacting samples twice or three times will produce a lot of broken kernels, which further increases the workloads of postprocessing. As a result, for the same set of samples, the test number is once as the optimum time. Each experiment needs to be repeated three times and taking the average value. On the one side, the HANDY operated at a lower speed cannot cause detectable damage to kernels. On the other side, higher speed will cause serious damage to kernels and increase the number of broken kernels. As shown in Table 1, the speed less than 1500 r/min and more than 2100 r/min can produce little and massive broken kernels, respectively. Both too little or too much-broken kernels be caused are unacceptable for the experiments, and the detail is discussed in the chapter of “Test for establishing optimum operating parameters of HANDY”. As a result, further experiments focus on the speed range of 1500–2100 r/min. Three centrifuge discs types of straight, curved and oblique are chosen for further testing because they can affect the moving direction of the kernels [24, 25]. Therefore, the experimental factors and variables in this research are: Centrifugal speed = 1500, 1800, 2100 r/min (23.6, 28.3, 33 m/s); Centrifuge discs type = straight, curved and oblique; Testing times = once.

## Results and discuss

The test results obtained in this study are reported in three main parts. The first part discusses the optimum parameters of the HANDY. The second part describes the results of the experiments which are designed to compare the effectiveness and applicability of BI and BS. BI and BS are utilized in evaluating the mechanical crushing resistance of kernels. The third part analyzes the applicability of the HANDY to evaluate the threshing quality of maize kernels.

### Working condition of HANDY

During the testing, a slight irregular vibration of the frame is observed. The impact part can generate a small noise though it is fixed to the ground by the rubber casters. The operation of the HANDY is not as laborious as traditional testers for measuring maize crushing resistance. Great flexibility in adjusting operation parameters is achieved with the HANDY. However, the device needs to improve its intelligence in picking un-sieve broken kernels.

### Test for establishing optimum operating parameters of HANDY

#### Peripheral speed of the centrifuge disc

**Fig.4:**
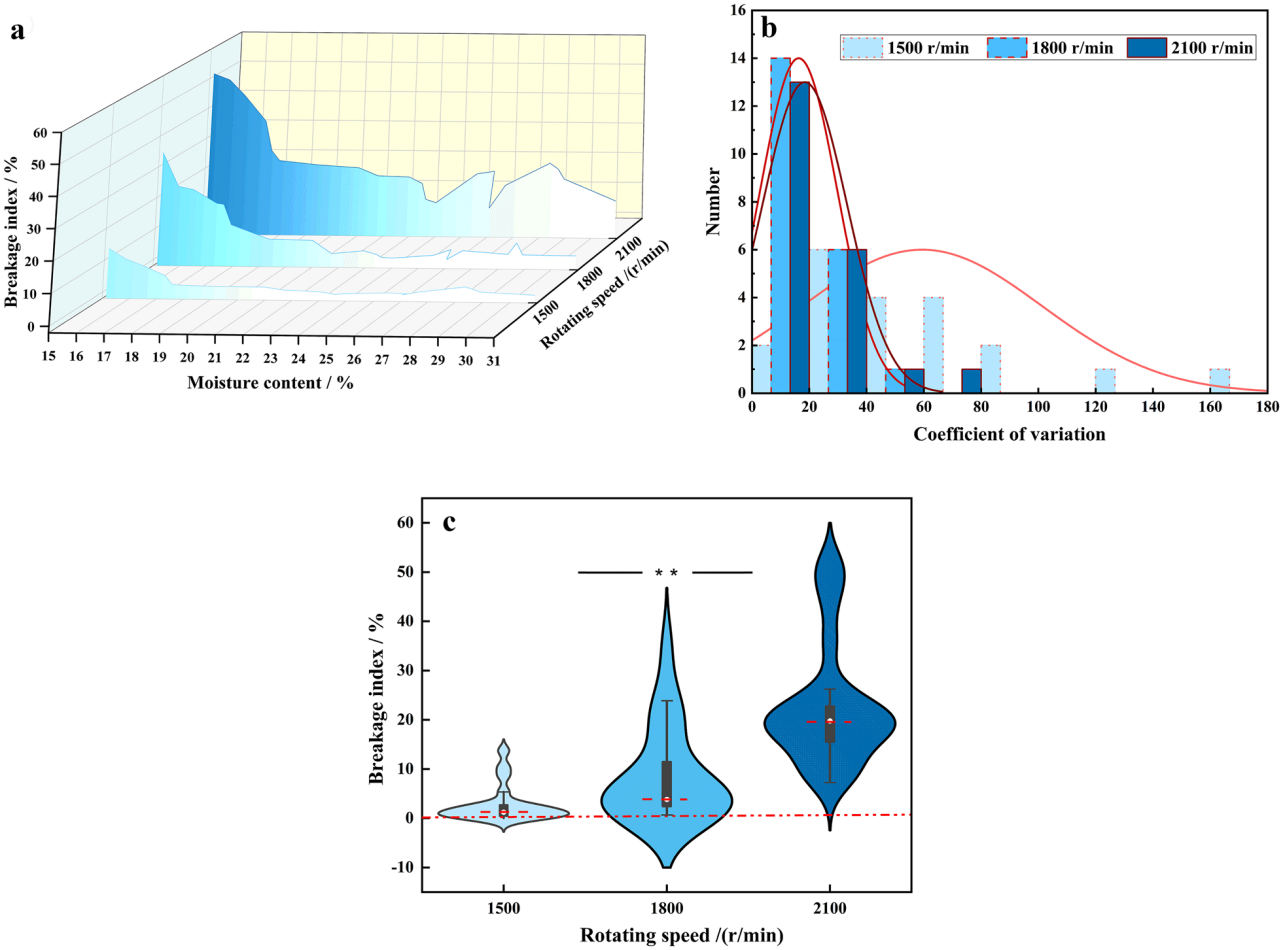
Results comparison of different speed to evaluate maize kernel

In order to ensure the device to have a stable performance and repeatable results, the tests using the straight type centrifuge disc are conducted at three peripheral speeds (23.6 m/s and 28.3 m/s and 33 m/s correspondings to 1500 r/min, 1800 r/min, and 2100 r/min rotating speed). The speed range has been determined by the baseline test result. The BI at three rotating speeds is depicted in Fig. 4a. The BI measured with three rotating speeds (1500 r/min, 1800 r/min and 2100 r/min) are 0–13.7, 1–34.7%, 7.6–51.7%, respectively. The average BI is 2.8, 8.5 and 22.7%, respectively. Statistical analysis of the BI shows a remarkable consistency in the results when the speed is different: the three curves of BI present a similar trend. Specifically, the BI decreased with the moisture content increased under the overall, which is almost in long-tailed distribution. In order to obtain the BI with smaller variability, both the variation of coefficient and distribution dispersion under different rotating speeds are discussed.

Figure 4b shows the coefficient of variation of the BI at different rotating speeds. The average coefficients of variation of the results are 56.6, 16.3, 19.1 when rotating speeds are 1500 and 1800 and 2100 r/min, respectively. Note that the BI is close to 0 when the rotating speed is 1500 r/min and the kernel moisture is more than 21%. In this case, small numerical changes of the BI can also have a significant effect on the coefficient of variation, resulting in low repeatability. This is the reason why the coefficient of variation of BI is larger when the rotating speed is 1500 r/min than others. As a result, the speed at 1500 r/min is too low for the HANDY operation. The coefficient of variation of the BI obtained at the speed of 2100 r/min is worse than those at the speed of 1800 r/min. This also shows that when the HANDY works at the rotating speed of 1800 r/min exhibited better repeatability and higher precision in discovering the kernels with different level of mechanical crushing resistance.

Figure 4c shows the distribution of the BI at different rotating speeds. When the rotating speed is 1500 r/min, 50% of the BI is less than 1.5%, which indicates that the BI has significant uneven distribution. This further shows that the HANDY operated at this speed causes less measurable damage to kernels. When the rotating speed is 1800 r/min, 57.1% of the BI is within 3%-7%, 50% of which are within 2–3%. The BI varied among different-moisture intervals, which ensure the continuity of BI. The HANDY works at the rotating of 2100 r/min can produce a substantial amount of damage to kernels that have various crushing resistance.

In addition to the above discussions, the time to pick the un-sieved broken kernels also needs to be considered. When the rotating speed is lower than 1500 r/min, the impact energy cannot be transmitted from the centrifugal disc to the kernels sufficiently. As a result, few kernels are broken completely and it is hard to pick them out. On the contrary, when the rotating speed is higher than 2100 r/min, it will take about additional 2 minutes to pick the broken kernels out. It even produces massive maize flour or juice. However, when the rotating speed is about 1800 r/min, a number of the kernels are broken with obviously broken characteristics and then easy to be picked. Moreover, the time to pick broken kernels is acceptable. Thus, when the speed is around 1800 r/min, the test results are conducive to evaluate the crushing resistance of kernels.

#### Type of the centrifuge disc

**Fig.5:**
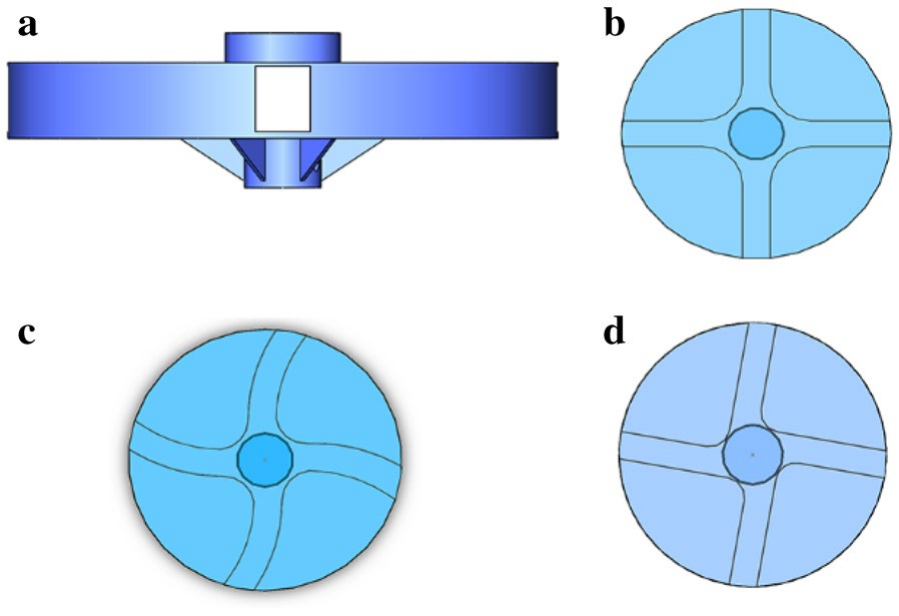
Type of disc

From a machine design perspective, the design objective of the centrifugal disc should make all the kernels be subjected to identical impact, and produce little random splatter of kernels. From the angle of kinematics, the type of discs can affect the magnitude and direction when kernels departing from the discs [26]. Thus, the objective of the analysis is to find an optimal centrifuge disc type. Three types of centrifuge disc (straight, curved and oblique) are selected and designed in this study (Fig. 5). Tests are conducted at rotating speeds of 1800 r/min by the HANDY.

**Fig.6:**
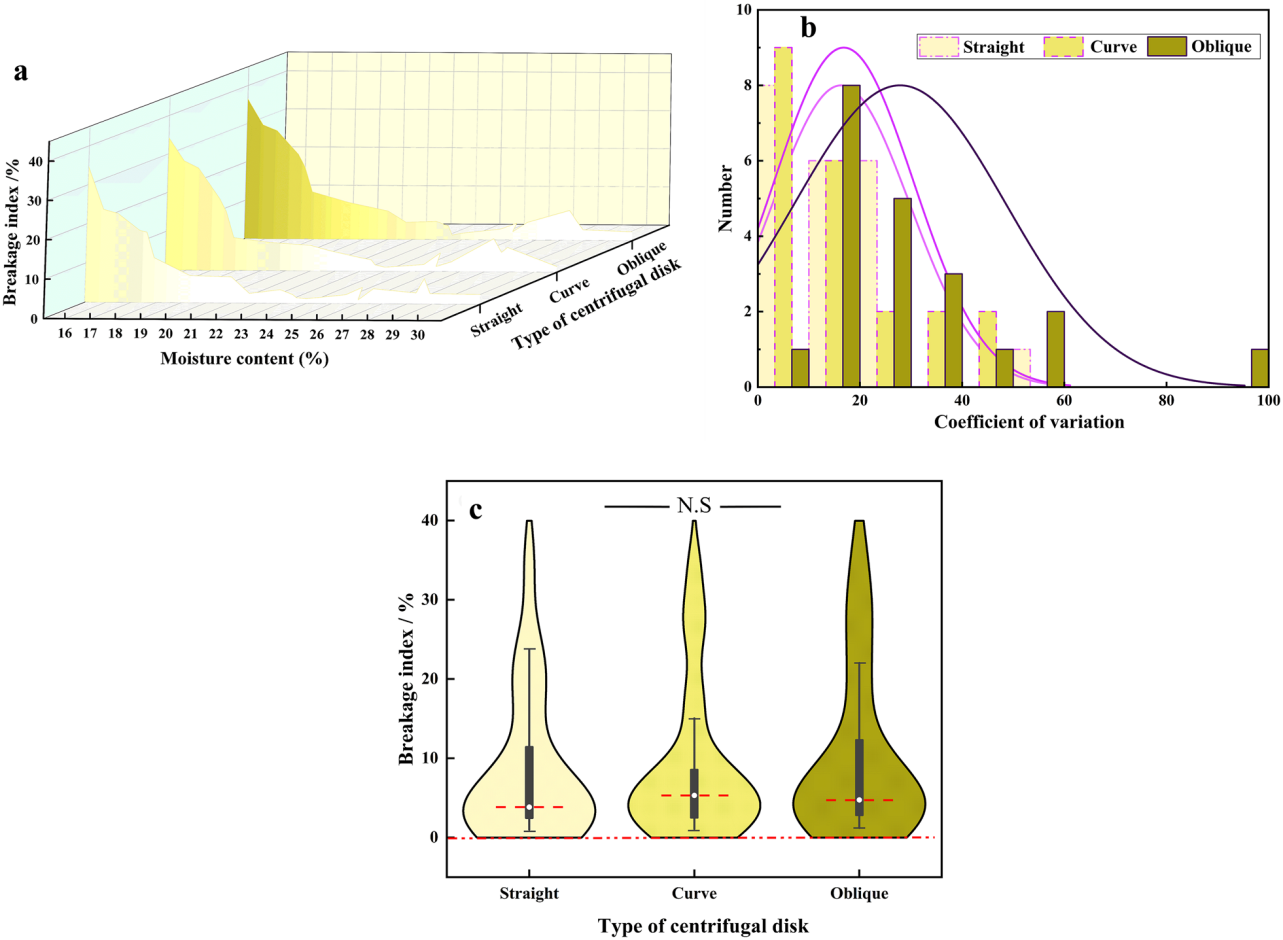
Results comparison of the different type of centrifuge disc to evaluate maize kernel

Figure 6a shows the value of BI when using different types of centrifugal discs. For all the tested maize varieties, the BI for different types of centrifuge discs (straight, curved and oblique) are within 1–34.7%, 1.2–36.6%, 1.1–34.2%, respectively. The BI decreases with the increase in moisture content. Note that, when the moisture content increases to about 23%, the BI decreases to a minimum. When the moisture content continues to increase, the BI changes within a small range.

The coefficient of variation and the distribution dispersion of the BI measured with different centrifuge disc types are discussed. As Fig. 6b shows, the curved disc produces the BI with a lower coefficient of variation compared to the straight and oblique disc. The average coefficient of variation for the straight and oblique disc is 1 and 1.7 times greater than that shown by the curved disc, respectively.

**Fig.7:**
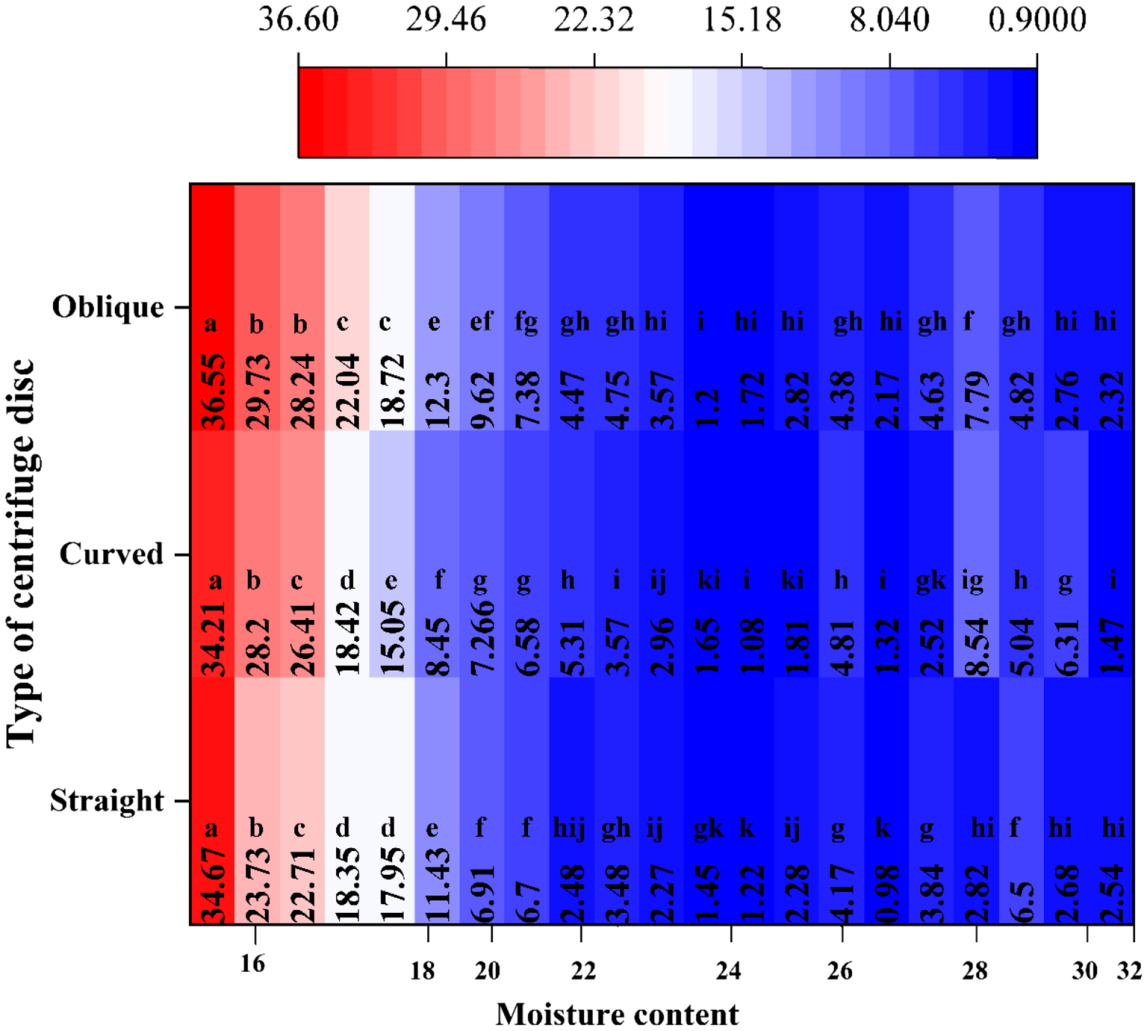
Difference examination of BI between moistures by use different disc type

Figure 6c shows the distribution of the BI for a different type of centrifugal discs. The SPSS 23.0 is used, the influence of centrifugal disc type on BI is analyzed through two methods: descriptive statistics analysis and difference testing. There is no significant difference in data distribution among the three centrifugal discs. This means that for the kernels with the same crushing resistance, all centrifugal discs can cause a considerable amount of damage to them. However, the results of the different tests show that more sensibility for the same test kernel samples by using the curved centrifugal disc (Fig. 7). This further shows that in comparison with the straight and oblique centrifugal discs, the curved type has superior sensitivity and is suitable for assessing to mechanical crushing resistance of maize kernel. It is more effective to distinguish the maize varieties with a small difference in crushing resistance. Therefore, it is appropriate to choose the curve type as the optimum centrifugal disc.

### Repeatability of results

**Fig.8:**
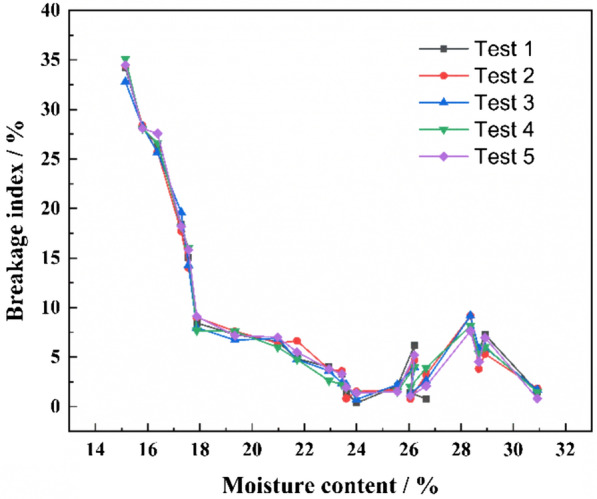
BI versus moisture content of the maize kernel during the repeatability test for the HANDY (Speed is 1800 r/min; Type of centrifugal disc is curved)

The plots of BI moisture content for the maize kernel (Fig. 8) show that the graphs for the five replicate tests are similar. The maximum difference of BI within any set is 1.2% which shows that the HANDY can produce repeatable results (speed: 1800 r/min, disc type: curved type).

### Breakage Susceptibility test results

**Fig.9:**
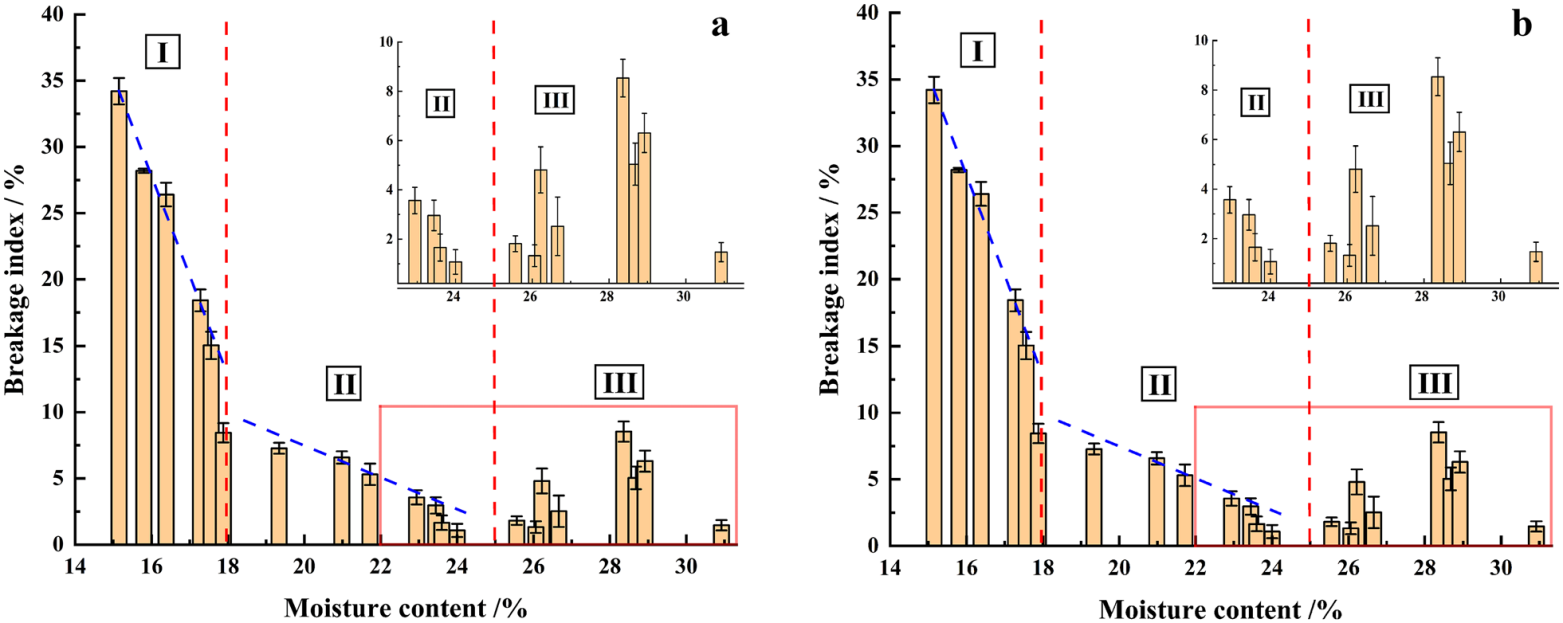
Results of breakage susceptibility and breakage index

Figure 9a shows the results of the Breakage Susceptibility tests. As expected, the results follow the normal breakage behavior of kernels. That is the BS of kernels decreases as the moisture content increases. When the BS is at maximum value, the maize kernels show higher mechanical crushing resistance. The change rules of BS of maize kernels obtained by HANDY are similar to those obtained by using the Stein Crush resistance tester, Wisconsin Tester, and Centrifugal Corn Crush resistance tester in previous studies [27–30].

### HANDY test results

The rotating speed of HANDY is 1800 r/min with the curved centrifugal disc. The BI was obtained for the same maize kernels. As shown in Fig. 9b, the change rules of BI obtained by HANDY tests and BS obtained by the Breakage Susceptibility tests are similar. For both the Breakage Susceptibility tests and the HANDY tests, the moisture content of the kernel is used as a variable. With the increasing of moisture content, the overall changing rules of BI and BS can be divided into three stages: stage I, stage II and stage III. The moisture content ranges of the three stages are 14–18%, 18–25%, and 25–31%.

For stage I (14–18%), stage II (18–25%), and stage III, both BS and BI drop sharply, drop slowly and keep stable with the increase of moisture content, respectively. An obvious difference between BS and BI in stage III is observed. Specifically, the results of BI increase to the maximum value and then decreases with the further increases in moisture content [31, 32]. In contrast, the BS in stage III is reduced close to zero. Consider the energy absorption capability, the wet kernels are higher than dry ones, the kernels achieve greater flexibility at high moisture, thus making the kernels absorb more deformation energy before crack [33, 34]. An increase in the moisture content of maize kernels increases the coefficient of friction and apparent cohesion of the kernel’s surface while making the surface softer and more deformable [35]. Therefore, when the moisture content of kernels is high, it cannot be neglected that numerous kernels are split into parts but still connected by a seed coat. The geometric mean diameter is 8.17 ± 0.37 mm, which is larger than the diameter of the round hole sieve of 12/64 inches (4.76 mm). As a result, those broken kernels connected by the seed coat cannot pass the circular sieve, resulting in the BS reduced close to zero in stage III (as shown in Fig. 9a). However, in the HANDY tests, BI shows a bell-shaped curve in stage III (as shown in Fig. 9b). As a result, compared with the BS, the BI can effectively reflect and evaluate the mechanical crushing resistance of the kernels at this stage.

### Results of mechanical threshing tests

Generally, mechanical damage is induced by impact during harvesting which can debase the quality and shortens the storage period of maize kernels [36, 37]. In order to prove that the HANDY can be used to predict the impact damage severity of maize during harvesting, the mechanical threshing test of maize ear is carried out. The HANDY test, meanwhile, is conducted at the optimum parameters.

**Fig.10:**
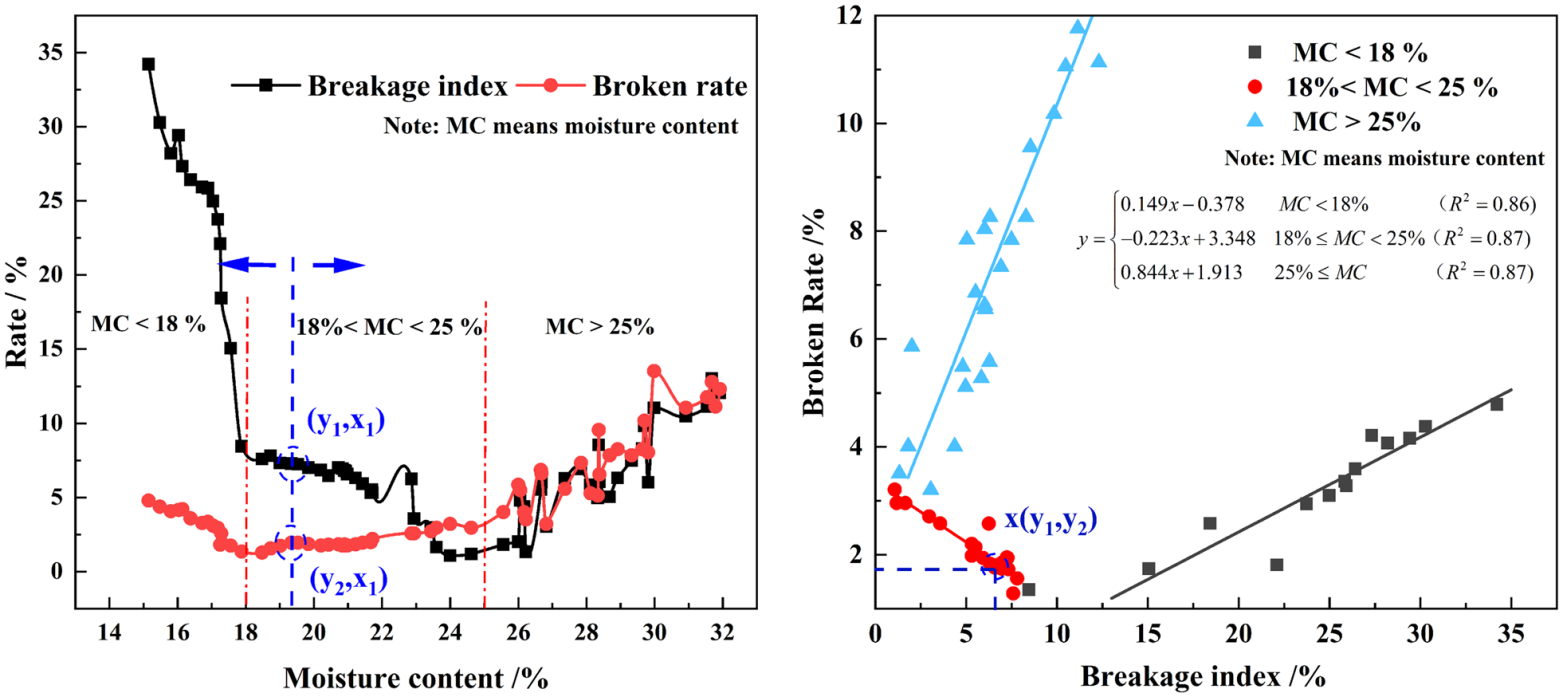
The results of broken rate and the breakage index of kernels

The results of BR and BI are shown in Fig. 10a. For all the tested hybrids, the ranges of the BR and BI are 1.3–13.5%, 1.1–34.2%, respectively. Figure 10a indicates that the relationship between the BR and the BI is diverse in three moisture content ranges. When the moisture content is less than 18%, both BR and BI decreased with the increase of moisture content, but the decreasing rate of BI is higher than BR. When the moisture content is 18%-25%, the BI continuously decreasing but the BR is increasing. When the moisture content is more than 25%, the change regularity of BR and BI is similar and increasing overall. In aggregate, with the increase of kernel moisture content, the broken rate first decreased and then increased, which is close to the result obtained before [4].

For maize kernel materials, the lower the moisture content, the higher the hardness and brittleness [14]. Therefore, the mechanical characteristics of kernels are brittle and hard in stage I, which makes them more likely to break into small pieces under the impact forces. In this stage (MC < 18%), for the HANDY test, each kernel will be accelerated and impacted with the shell, and then broken into small pieces. On the contrary, in the mechanical threshing test, the ears are impacted by threshing elements, most of the kernels are threshed from the mandrel. In this condition, the number of kernels is impacted by threshing elements is limited, which resulting the BR is small. Therefore, in stage I, with the increase of moisture content, the increase rate of BI is higher than that of BR. In stage III (MC > 25%), the kernels show plasticity, high elasticity and flexibility. An increase in moisture content increases the coefficient of friction and apparent cohesion of the kernel’s surface while making the surface softer and more deformable. For ear, with the increase of moisture content, the narrow wedge-shaped space formed by the transverse space between kernels decreased, the kernels contacted closely, the interaction force between kernels increased. Besides, with the increase of the number of kernels, the greater the compressive strength of the kernels is, the greater the threshing capacity is required. Therefore, in stage III, with the increase of moisture content, the increase rate of BI is lower than that of BR. In stage II (18% < MC < 25%), the mechanical characteristics of kernels are between that in stage I and stage III. For the mechanical threshing test, the connection between kernel and mandrel is very complex, which results in the BR changeless in this stage. However, for the HANDY test, BI generally increased in magnitude with an increase in moisture content.

Figure 10b shows the relationship between the BR and the BI which eliminates the information of kernel moisture compared with Fig. 10a. The plot procedure is as follows: First, select a single moisture content of kernel on both curves of BI and BS (denoted by the straight line in Fig. 10a). The intersection of this straight line with the BI curve becomes the x-coordinate and the intersection of the straight line with the BS curve becomes the y-coordinate of the Fig. 10b. Plot this point in a separate graph (point X in Fig. 10b), which represents the relationship between the BR and the BI and no longer possesses a kernel moisture element. Repeat this process for each kernel moisture to construct the remainder of Fig. 10b. As a result, the BI (independent variable) is used to generate subsection linear regression functions that could be used to predict BR (dependent variable) achieved using the HANDY. The equation is:

4 $$y = \left\{ {\begin{array}{*{20}c} {0.149x - 0.378} & {MC < 18\% } & {(R^{2} = 0.86)} \\ { - 0.223x + 3.348} & {18\% \le MC < 25\% } & {(R^{2} = 0.87)} \\ {0.844x + 1.913} & {25\% \le MC} & {(R^{2} = 0.87)} \\ \end{array} } \right.$$

The R^2^ of the regression model at three moisture content is 0.86, 0.87, and 0.87, respectively, which further illustrates the BI prove capable of explaining on average about 86.7% of the BR of maize kernel in threshing.

**Fig.11:**
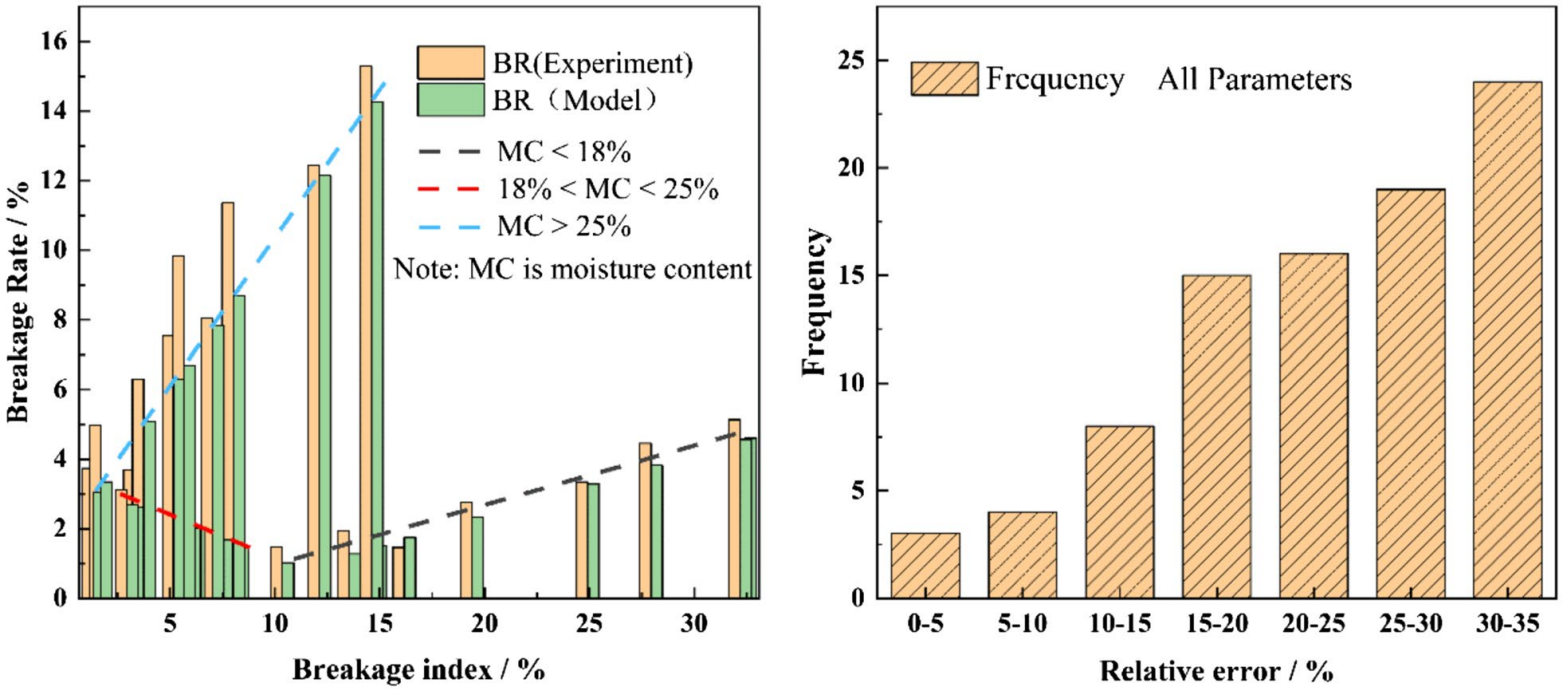
Evaluation of approximation accuracy level of the subsection linear regression model

These values indicate that the subsection linear regression model may be considered satisfactory, however, it is necessary to check the linear regression model in Eq. (4) to evaluate whether it can provide an acceptable approximation or not. The approximation precision level of the linear regression model is evaluated through the calculation of relative errors between the results obtained from the HANDY and threshing tests. The evaluation results are given in Fig. 11. As expected, the results show that the BR predicted values and the measured ones are in good conformity at three kernel moisture ranges (Fig. 11a). As shown in Fig. 11b, it’s seen that the range of relative error is calculated between 2 and 32.8%. The average relative error for the three moisture ranges is 11.1, 23.7 and 17.86%, respectively. A relative error of less than 20% is observed for 15 out of 24 experiments (i.e. for 62.5% of experiments). As a result, considering the variability of physiological characters in maize kernel the linear regression model the coefficient of determinations of these regression equations are satisfactory.

### Evaluation of maize kernel crushing resistance

**Fig.12:**
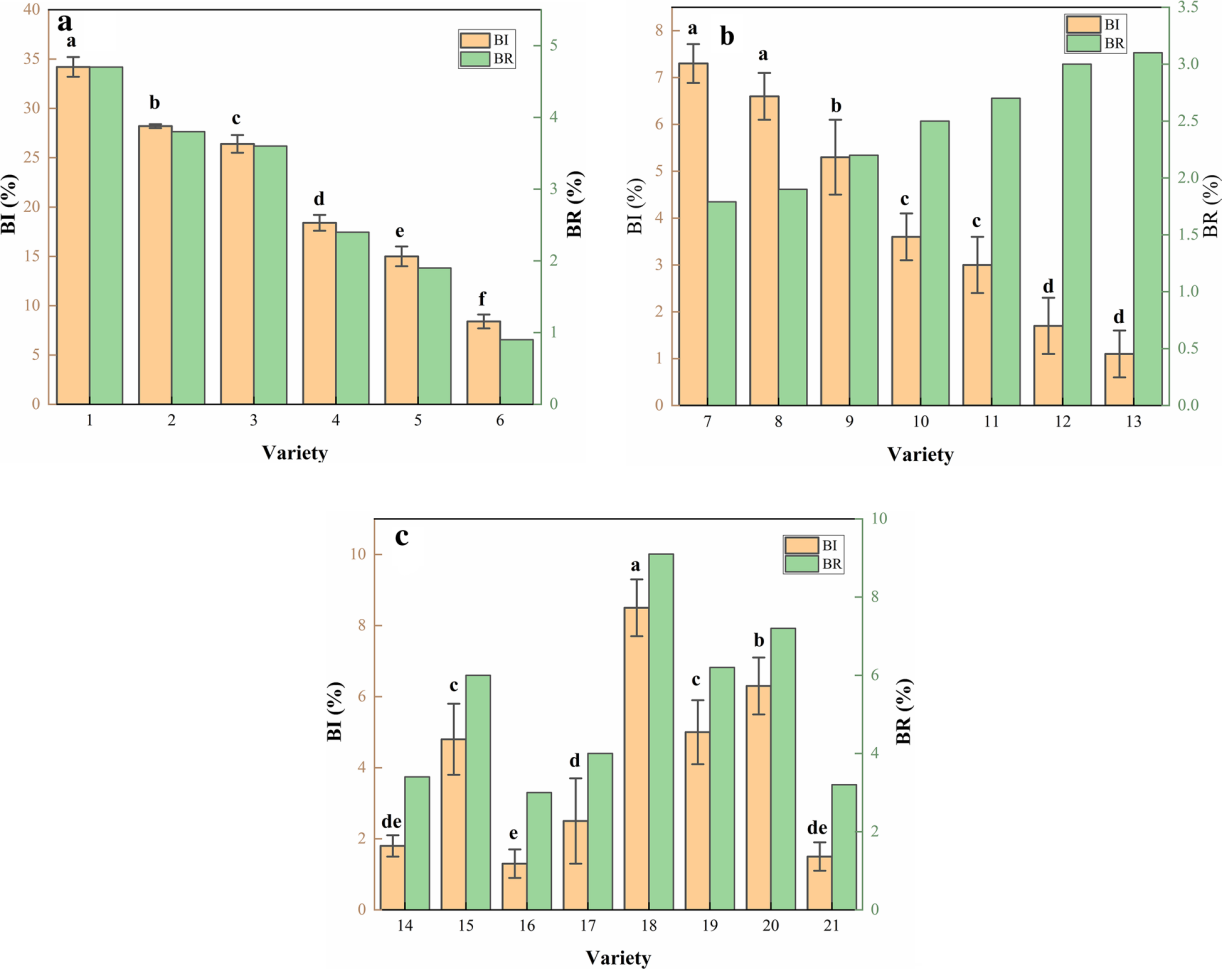
Breakage data of maize varieties under optimum testing conditions (centrifugal disc type: curved, speed: 1800 r/min)

In this study, BI is found to have remarkable correlativity with crushing resistance and be treated as an index to represent crushing resistance for maize kernel. It is not negligible that different cultivars expressed different sensitivity to impact force, this is related to their different capability of crushing resistance. Thus, the BI and BR of maize kernels under different cultivars and test parameters are discussed in this Sect. 21 test varieties are classed into three groups by different moisture contents. The BI is tested by HANDY at the decided working parameters (curved, 1800 r/min). The BR of the test verities is estimated using Eq. 4. The breakage data are shown in Fig. 12.

**Fig.13:**
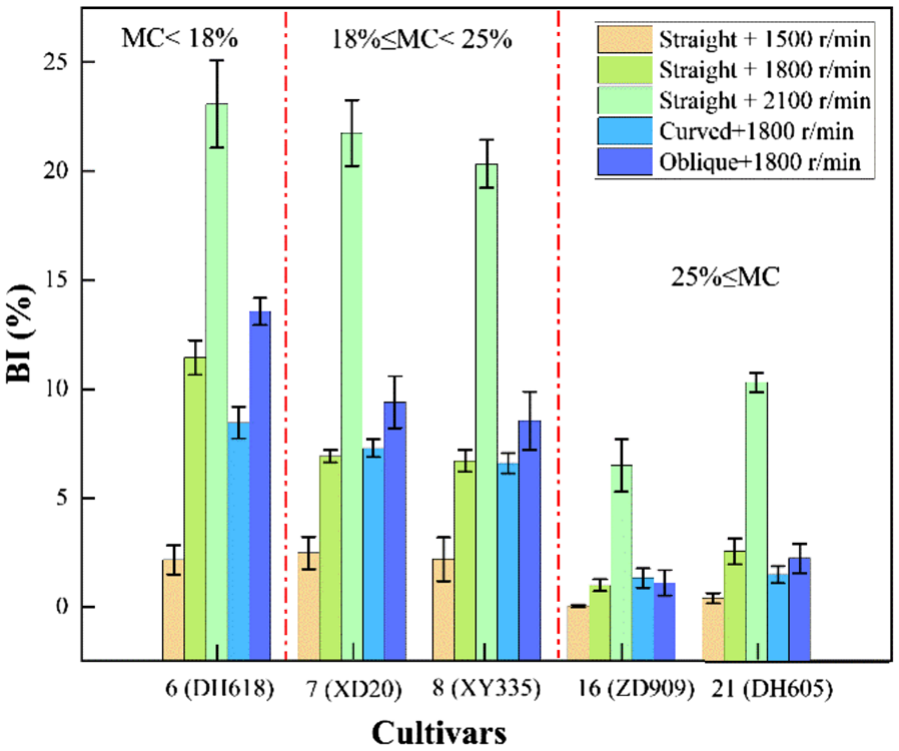
Breakage data of maize varieties under different testing conditions

Five maize cultivars are identified with higher damage resistance among 21 tested candidate varieties. Cultivars 6 (DH618) at group one, 7 (XD20) and 8 (XY335) at group two,16 (ZD909) and 21 (DH605) at group three have the lowest BR of 0.9%, 1.7%, 1.9%, 3.0% and 3.2%, respectively. Those cultivars have better damage resistance than the others in the same group when suffering the same load. Breakage data of selected cultivars under different testing conditions are shown in Fig. 13.

As shown in Fig. 13, when using the curved type centrifugal disc, with the increase of rotation speed, the BI increased obviously. It can also be found that the increments of BI of varieties 6 (DH618), 7 (XD20), and 8 (XY335) are similar under different speeds. For cultivars 16 (ZD909) and 21 (DH605), the BI of the oblique centrifugal disk is greater than that of the curved type.

## Conclusion

This work is undertaken to develop a device called HANDY for assessing resistance to mechanical crushing of maize kernels. Imitating the loading model (impact) of the mechanical threshing of kernels, HANDY is designed based on centrifugal acceleration, which is used to provide acceleration power for kernels. For obtaining results with small variability and high repeatability, the curve type centrifugal disc and the speed of about 1800 r/min can be chosen. Compared with the traditional Breakage Susceptibility test, HANDY has greater sensitivity in determining the influence of higher moisture content on the measurement of crushing resistance. A linear regression model is developed to relate the HANDY test results to the mechanical threshing quality, with an average R^2^ of 0.9. Five maize cultivars are identified with higher damage resistance among 21 tested candidate varieties. The HANDY, moreover, this prototype is flexible and can be modified for testing many other grains.

## Supplementary Information

**Additional file 1.** Structural parameters of HANDY.

**Additional file 2.** Maize samples.

**Additional file 3.** Photograph of HANDY.

## Data Availability

The datasets used and/or analyzed during the current study are available from the corresponding author on reasonable request.

## Abbreviations

BI: Breakage index (%)
BS: Breakage susceptibility (%)
BR: Breakage rate (%)
