# Supplementary material for: HANDY: a device for assessing resistance to mechanical crushing of maize kernel
Source: Plant Methods. 2021 Apr 26;17:44. doi: 10.1186/s13007-021-00729-2 (PMC8074406; doi:10.1186/s13007-021-00729-2)
Supplement: Supplementary file 1 — Additional file 1. Structural parameters of HANDY. [file 13007_2021_729_MOESM1_ESM.docx]

**Additional file**

**Additional file 1. Structural parameters of HANDY.**

| Parameter | Value |
| --- | --- |
| Diameter of shell upper /mm | 300 |
| Taper of the lower part of the housing /° | ＞70 |
| Diameter of centrifugal disk /mm | 240 |
| Width and height of guide rails /（mm×mm） | 20 × 20 |
| Speed range of motor /（r·min^-1^） | 0 ~ 1200 |
| Hole diameter of the circular sieve / inch | 12/64 |
