# Supplementary material for: HANDY: a device for assessing resistance to mechanical crushing of maize kernel
Source: Plant Methods. 2021 Apr 26;17:44. doi: 10.1186/s13007-021-00729-2 (PMC8074406; doi:10.1186/s13007-021-00729-2)
Supplement: Supplementary file 2 — Additional file 2. Maize samples. [file 13007_2021_729_MOESM2_ESM.docx]

**Additional file 2. Maize samples.**

| Variety characteristics | No. | Varieties | Moisture Contend/% |
| --- | --- | --- | --- |
| Half-dent | 1 | WY964 | 16.38 |
|  | 2 | LP206 | 17.28 |
|  | 3 | LY31 | 17.56 |
|  | 4 | ZD909 | 19.34 |
|  | 5 | ND108 | 20.98 |
|  | 6 | LP208 | 21.92 |
|  | 7 | JY101 | 22.94 |
|  | 8 | SR999 | 23.60 |
|  | 9 | LY37 | 24.00 |
|  | 10 | XD20 | 26.06 |
|  | 11 | JD28 | 28.68 |
|  | 12 | JF339 | 30.92 |
|  | 13 | JNK728 | 28.92 |
| Dent | 14 | JN858 | 28.36 |
|  | 15 | DK517 | 26.66 |
|  | 16 | DH618 | 25.56 |
|  | 17 | DH605 | 15.15 |
| Flint | 18 | XY335 | 26.22 |
|  | 19 | JY1H | 23.44 |
|  | 20 | YD13 | 17.28 |
|  | 21 | JF223 | 15.80 |
