# Supplementary figures and images for: HANDY: a device for assessing resistance to mechanical crushing of maize kernel
Source: Plant Methods. 2021 Apr 26;17:44. doi: 10.1186/s13007-021-00729-2 (PMC8074406; doi:10.1186/s13007-021-00729-2)

**Additional file 3. Photograph of HANDY.**


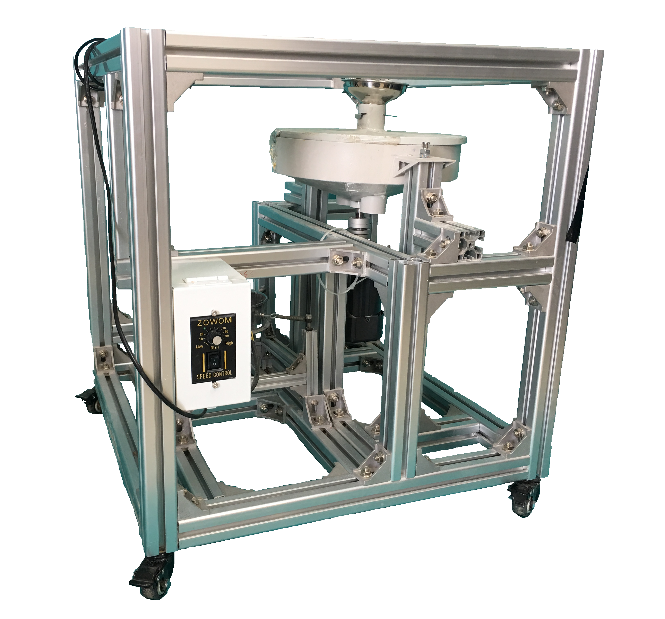

Supplement: Supplementary file 3 — Additional file 3. Photograph of HANDY. [file 13007_2021_729_MOESM3_ESM.docx]
